# Robotic surgery in obstetrics and gynecology: a bibliometric study

**DOI:** 10.1007/s11701-023-01672-1

**Published:** 2023-07-10

**Authors:** Gabriel Levin, Matthew Siedhoff, Kelly N. Wright, Mireille D. Truong, Kacey Hamilton, Yoav Brezinov, Walter Gotlieb, Raanan Meyer

**Affiliations:** 1 Lady Davis Institute for Cancer Research, Jewish General Hospital, McGill University, Quebec, Canada; 2 Division of Minimally Invasive Gynecologic Surgery, Department of Obstetrics and Gynecology, Cedars Sinai Medical Center, Los Angeles, CA USA; 3 The Dr. Pinchas Bornstein Talpiot Medical Leadership Program, Sheba Medical Center, Tel Hashomer, Ramat-Gan, Israel

**Keywords:** Bibliometrics, Citations, Gynecologic surgery, Research, Robot

## Abstract

We aimed to identify the trends and patterns of robotic surgery research in obstetrics and gynecology since its implementation. We used data from Clarivate’s Web of Science platform to identify all articles published on robotic surgery in obstetrics and gynecology. A total of 838 publications were included in the analysis. Of these, 485 (57.9%) were from North America and 281 (26.0%) from Europe. 788 (94.0%) articles originated in high-income countries and none from low-income countries. The number of publications per year reached a peak of 69 articles in 2014. The subject of 344 (41.1%) of articles was gynecologic oncology, followed by benign gynecology (n = 176, 21.0%) and urogynecology (n = 156, 18.6%). Articles discussing gynecologic oncology had lower representation in low- and middle-income countries (LMIC) (32.0% vs. 41.6%, p < 0.001) compared with high income countries. After 2015 there has been a higher representation of publications from Asia (19.7% vs. 7.7%) and from LMIC (8.4% vs. 2.6%), compared to the preceding years. In a multivariable regression analysis, journal’s impact factor [aOR 95% CI 1.30 (1.16–1.41)], gynecologic oncology subject [aOR 95% CI 1.73 (1.06–2.81)] and randomized controlled trials [aOR 95% CI 3.67 (1.47–9.16)] were associated with higher number of citations per year. In conclusion, robotic surgery research in obstetrics & gynecology is dominated by research in gynecologic oncology and reached a peak nearly a decade ago. The disparity in the quantity and quality of robotic research between high income countries and LMIC raises concerns regarding the access of the latter to high quality healthcare resources such as robotic surgery.

**Supplementary Information:**

The online version contains supplementary material available at 10.1007/s11701-023-01672-1.

## Introduction

Robotic surgery was introduced in 1985 to allow greater precision in surgery and this field has been developing since then [1, 2]. While originally driven by the U.S army and the National Air and Space Administration (NASA), seeking for means of telesurgery (performing a surgery by a geographically distant surgeon) [3], it soon evolved into a pivotal tool in modern surgery when precision is needed [4].

Cardiology, gynecology, and urology were the specialty forerunners of robotic surgery and dominated the early period of robotic surgery studies and research [5], later followed by other disciplines including spinal surgery and general surgery [6].

Robotic surgery and telesurgery can assist in minimizing inequity in access to surgical services among low- and middle-income countries (LMIC) [7, 8]. However, the introduction of robotic surgery to LMIC is hindered by high costs, both acquisition and maintenance, and for some part shortage of trained surgeons [9, 10].

In a recent scientific impact paper by the royal college of obstetricians and gynecologists (RCOG), it was stated that robotic surgery is a safe and effective surgical tool for women who have to undergo complex gynecologic surgery or have certain comorbidities [11]. Despite this clinical importance, the research span of robotic surgery in obstetrics and gynecology (OBGYN) is currently unknow.

A recent literature review of robotic surgery publications in 2001–2021 reported 3800 publications on the topic and concluded that robotic surgery research is still limited [6]. Of note, that analysis reported on publications in all medical disciplines, and included non-original research studies including meeting abstracts, letters, and editorial materials.

Bibliometric analysis can study the quality of research through the measurement of various parameters and point out trends in research and publications, thereby assisting in identifying unmet gaps of research and inequality [12, 13].

This study aims to identify the research trends and patterns of robotic research in OBGYN since the beginning of robotic surgery use.

## Materials and methods

### Sample creation

We used Clarivate’s “Web of Science” (WOS) to identify all relevant publications on robotic surgery in OBGYN [14, 15]. WOS is one of the leading citation search platforms. It was thoroughly studied and found to be more accurate than other platforms [14, 16]. The term “Robotic” was used based on the American Board of Obstetrics and Gynecology (ABOG) 2022 certifying examination topics list [17]. We restricted the query to document types labeled as “Articles” and “Reviews” under the WOS Category “Obstetrics Gynecology” and “Oncology”. Only articles with the terms “Robotic” or “Robot” in their title were included [18]. Articles were further independently manually reviewed by two researchers (R.M and G.L) to ascertain that the manuscript’s topic included robotic surgery in obstetrics and gynecology. Bibliometric data were obtained from the iCite database, a National Institutes of Health (NIH) database. The following bibliometric data were collected: average citations per year (CPY, calculated by as total number of citations divided by the number of years since publication); relative citation ratio (RCR, based on weighting the number of citations a paper receives to a comparison group within the same field); field citation ratio (FCR, calculated by dividing the number of citations an article has received by the average number of citations received by articles published in the same year and in the same research fields). WOS and iCite databases were queried on December 15th, 2022.

### Variables of interest

Each of the identified articles was evaluated for specific characteristics including the continent and country of origin of the corresponding author, country’s level of income as defined by the world bank by annual gross national income per capita [19], publishing journal, publication year, CPY, RCR, FCR, journal impact factor, subject matter, and study design. Journals’ impact factor was defined according to the 2022 Clarivate’s Journal Citation Report. Study design was categorized into the following: retrospective, prospective, case report, review, case series, randomized controlled trial, video article, and meta-analysis. We defined high CPY as the 90th percentile of CPY and divided the cohort into two groups: high CPY (≥ 90th percentile) and low CPY (< 90th percentile). The 90th percentile of CPY was calculated to be 6.6. We further divided the cohort into two groups by the year of publication, below the median (early period) and above the median (2015)—late period.

As the data used for this study are publicly available and do not include protected health information, Institutional Review Board review and approval was not required.

### Statistical analysis

Statistical analyses were performed using SPSS version 29. We performed a descriptive analysis using a chi square test and Fisher exact test for categorical variables and a Wilcoxon rank-sum test for nonparametric continuous variables. Multivariable regression analysis was conducted to identify independent parameters associated with a high CPY. The regression analysis model included all factors that were found to be statistically significant in the univariate analysis. A 2-sided p value < 0.05 was considered statistically significant.

### Ethics approval

This is an observational study. The Cedars-Sinai Research Ethics Committee has confirmed that no ethical approval is required.

## Results

**Fig. 1:**
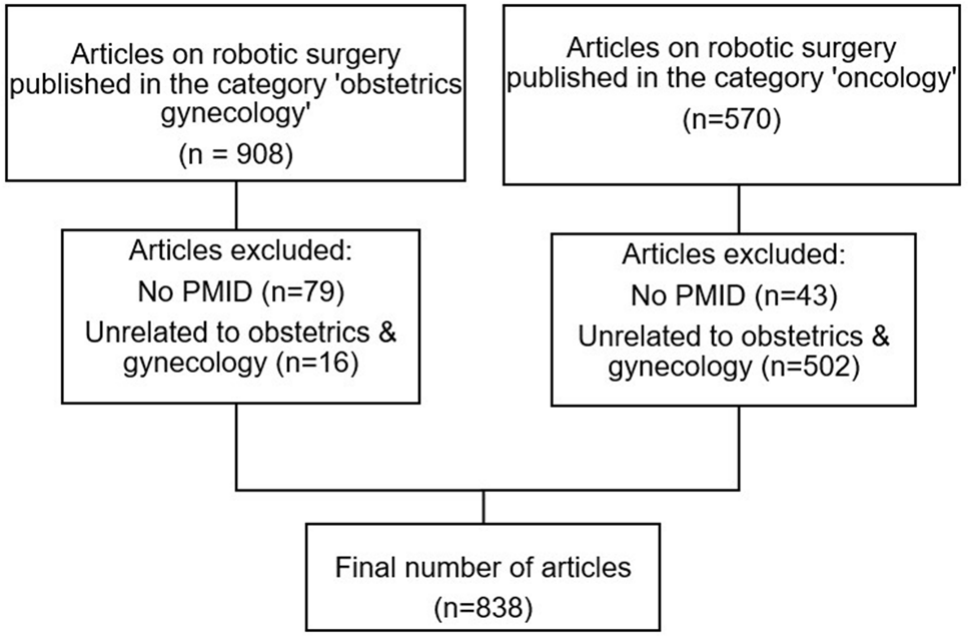
Study flow diagram. *PMID* PubMed identifier number

**Fig. 2:**
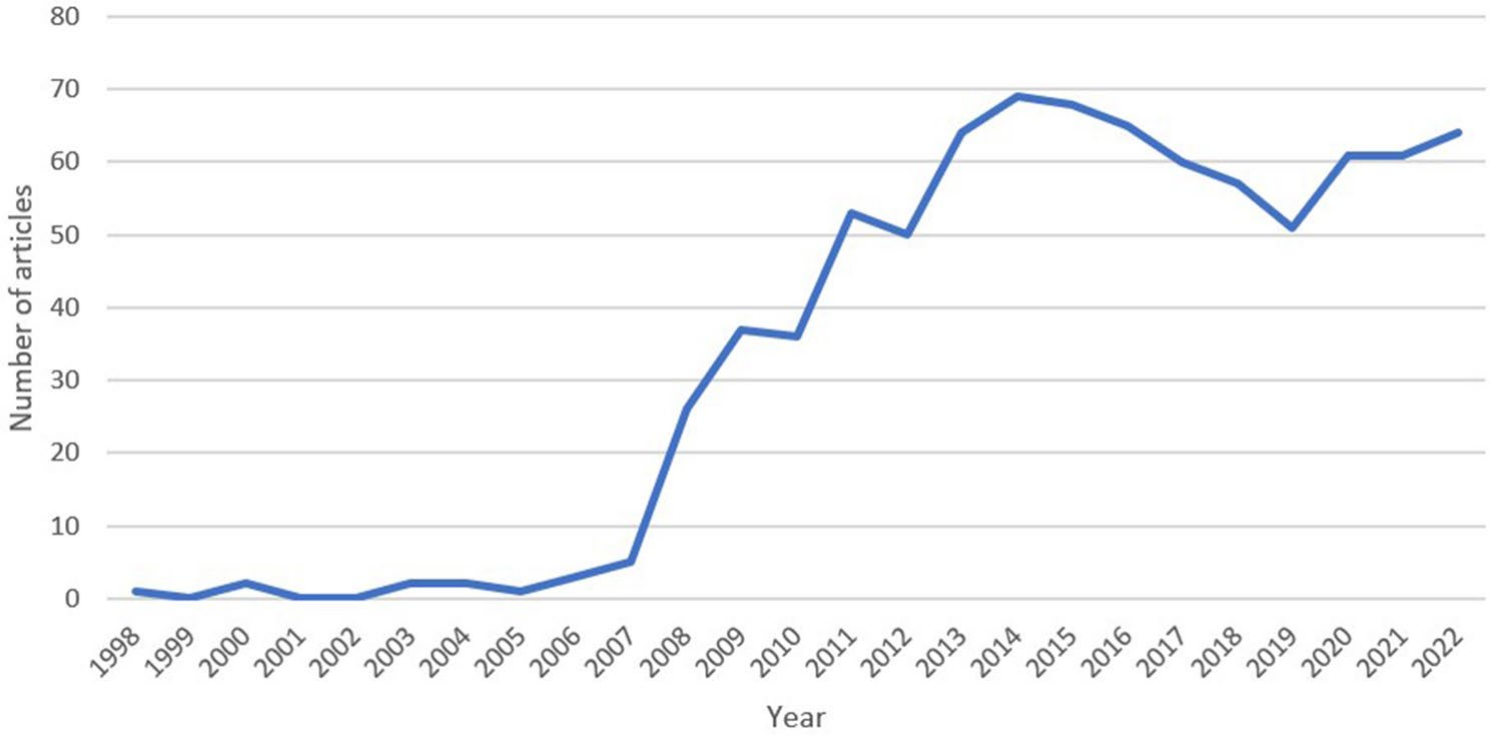
Number of articles published by year

A total of 1478 publications were retrieved from the search. We excluded 122 articles that did not have a PubMed identifier number (PMID) and 518 articles discussing robotic analysis unrelated to OBGYN. Finally, 838 articles were included in the analysis (Fig. 1). The first OBGYN robotic surgery article titled “Robotically assisted laparoscopic microsurgical uterine horn anastomosis” [20] was published in 1998. The median year of publication was 2015 [interquartile range (IQR) 2012–2019]. The number of publications per year has constantly increased reaching a peak of 69 articles per year in 2014 (Fig. 2). The 5-year period with the highest number of articles was 2013–2017 with 326 (38.9%) publications. The greatest proportional increase was between 2003–2007 and 2008–2012, with a 15.5-fold increase in the number of articles published (202 vs. 13, Figure S1).

### Country of origin

Most publications were of North American origin (n = 485, 57.9%, Table 1) predominantly from the U.S (n = 461, 55.0%), followed by European origin (n = 218, 26.0%) leaded by France and Italy (n = 49, 5.8%, n = 47, 5.6%, respectively). Overall, 788 (n = 94.0%) publications were from countries categorized as high-income countries. There were no publications from low-income countries and there were 50 publications (6.0%) from LMIC.

**Table 1:** Characteristics of included articles

Characteristics	n = 838, n (%)
Continent	
North America	485 (57.9%)
Europe	218 (26.0%)
Asia	123 (14.7%)
Australia	5 (0.6%)
South America	6 (0.7%)
Africa	1 (0.1%)
Countries’ level of income	
High	788 (94.0%)
Medium high	41 (4.9%)
Low medium	9 (1.1%)
Low	0 (0%)
Country	
United States	461 (55.0%)
France	49 (5.8%)
Italy	47 (5.6%)
South Korea	39 (4.7%)
Sweden	34 (4.1%)
Canada	24 (2.9%)
Turkey	22 (2.6%)
Taiwan	17 (2.0%)
Other	145 (17.3%)
Journal	
Journal of Minimally Invasive Gynecology	151 (18.0%)
Gynecologic Oncology	105 (12.5%)
International Journal of Gynecological Cancer	60 (7.2%)
International Urogynecology Journal	60 (7.2%)
Female Pelvic Medical Research	55 (6.6%)
American Journal of Obstetrics and Gynecology	38 (4.5%)
Obstetrics and Gynecology	37 (4.4%)
Archives of Gynecology and Obstetrics	24 (2.9%)
Acta Obstetrica Gynecologica Scandinavica	21 (2.5)
European Journal of Gynecology Obstetrics and Reproductive Biology	21 (2.5)
Taiwan Journal of Obstetrics and Gynecology	19 (2.3%)
Fertility Sterility	17 (2.0%)
Others	230 (27.4%)
Publication Year	2015 [2012–2019]
Total citations	10 [3–27]
Citations per year	1.9 [0.7–3.8]
Relative citation ratio	0.9 [0.3–1.9]
Field citation ratio	3.0 [2.5–3.8]
Impact factor	4.3 [1.9–5.3]
Subject	
Oncology	344 (41.1%)
Benign gynecology	176 (21.0%)
Urogynecology	156 (18.6%)
General	94 (11.2%)
Endometriosis	27 (3.2%)
Technique	25 (3.0%)
Fertility	8 (1.0%)
Obstetrics (cervical cerclage)	8 (1.0%)
Methodology	
Retrospective	394 (47.0%)
Prospective	177 (21.1%)
Case reports	79 (9.4%)
Review	93 (11.0%)
Case series	44 (5.3%)
Randomized controlled trial	30 (3.6%)
Video	20 (2.4%)
Meta-analysis	1 (0.1%)

Data are number (%) or median (interquartile range)

### LMIC context

In a comparison of publications from LMIC versus high income countries (Table 2), all medians of bibliometric scores were lower in LMIC publications. The median year of publication for LMIC publications was more recent (2017 vs. 2015, p = 0.002).

**Table 2:** Characteristics of included articles- low- and middle-income versus high-income countries

Variable	Low and middle income (n = 50)	High income (n = 788)	p value
Citations per year	0.6 [0–1.9]	2.0 [0.8–3.9]	< 0.001
Relative citation ratio	0.3 [0–1]	0.9 [0.3–1.9]	< 0.001
Field citation ratio	2.5 [1.8–3.3]	3.0 [2.5–3.9]	< 0.001
Total citations number	3 [0–11]	11 [3–29]	< 0.001
Publication year	2017 [2015–2020]	2015 [2012–2019]	0.002
Journal’s impact factor	1.9 [0–4.3]	4.3 [1.9–5.3]	< 0.001
Subject			< 0.001
Benign	20 (40.0%)	156 (19.8%)	
Endometriosis	4 (8.0%)	23 (2.9%)	
Fertility	2 (4.0%)	6 (0.8%)	
General	2 (4.0%)	92 (11.7%)	
Obstetrics	0 (0%)	8 (1.0%)	
Oncology	16 (32.0%)	328 (41.6%)	
Technique	1 (2.0%)	24 (3.0%)	
Urogynecology	5 (10.0%)	151 (19.2%)	
Continent			< 0.001
North America	0 (0%)	485 (61.5%)	
Europe	0 (0%)	218 (27.7%)	
Asia	43 (86.0%)	80 (10.2%)	
Australia	0 (0%)	5 (0.6%)	
South America	6 (12.0%)	0 (0%)	
Africa	1 (2.0%)	0 (0%)	
Methodology			< 0.001
Retrospective	24 (48.0%)	370 (47.0%)	
Prospective	2 (4.0%)	175 (22.2%)	
Case report	11 (22.0%)	68 (8.6%)	
Review	4 (8.0%)	89 (11.3%)	
Case series	8 (16.0%)	36 (4.6%)	
Randomized controlled trial	0 (0%)	30 (3.8%)	
Video	1 (2.0%)	19 (2.4%)	
Meta-analysis	0 (0%)	1 (0.1%)	

Data are number (%) or median (interquartile range)

Subjects of research that had higher representation in LMIC were benign gynecology [20 (40.0%) vs. 156 (19.8%)], endometriosis [4 (8.0%) vs. 23 (2.9%)], and fertility [2 (4.0) vs. 6 (0.8%)]. The types of publications that had higher representation in LMIC were case reports [11 (22.0%) vs. 68 (8.6%)], and case series [8 (16.0%) vs. 36 (4.6%)].

### Research subject and methodology

**Fig. 3:**
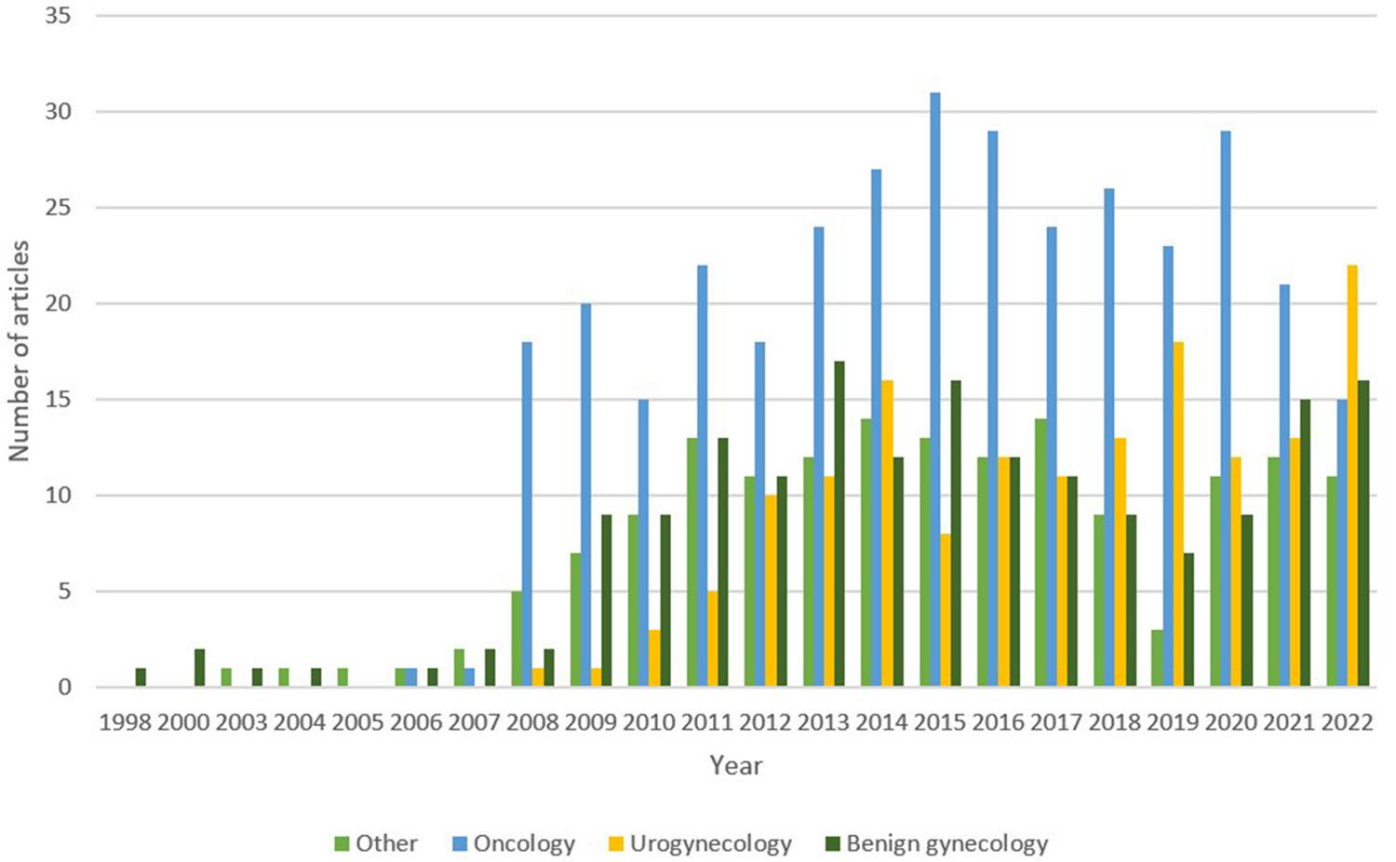
Number of articles published by year according to surgical field

Most publications were in the field of gynecologic oncology (n = 344, 41.1%), followed by benign gynecology (n = 176, 21.0%) and urogynecology (n = 156, 18.6%) (Table 1, Fig. 3). Most studies were retrospective (n = 394, 47.0%), followed by prospective (n = 177, 21.1%) and case series/reports (n = 123, 14.7%). RCT comprised 3.6% (n = 30) of all publications.

### Publishing journals

The leading publishing journal was the Journal of Minimally Invasive Gynecology, with 151 (18.0%) publications, followed by two gynecologic oncology journals (Gynecologic Oncology—n = 105, 12.5%, International Journal of Gynecological Cancer—n = 60, 7.2%), and two urogynecology journals (International Urogynecology Journal—n = 60, 7.2%, Female Pelvic Medical Research—n = 55, 6.6%, Table 1).

### Bibliometrics

The median Impact factor was 4.3 [1.9–5.3], with a median of 10.0 citations [3.0–27.0] and a CPY of 1.9 [0.7–3.8]. In an analysis of high CPY vs. low CPY (Table 3), the following topics were associated with higher CPY: oncology 45 (53.6%) vs. 299 (39.7%), and fertility 4 (4.8%) vs. 4 (0.5%). Urogynecology was associated with low CPY: 7 (8.3%) vs. 149 (19.8%). Publications from North America were associated with higher CPY: 62 (73.8%) vs. 423 (56.1%), as well as randomized controlled trials: 7 (9.5%) vs. 22 (2.9%).

**Table 3:** Characteristics of included articles- high median number of citations per year versus low median number of citations per year

Variable	90th (n = 84)	< 90th (n = 754)	p value
Citations per year	9.4 [7.8–12.5]	1.6 [0.6–3.0]	< 0.001
Relative citation ratio	4.6 [3.6–5.7]	0.7 [0.2–1.5]	< 0.001
Field citation ratio	3.6 [3.1–4.5]	2.9 [2.4–3.8]	< 0.001
Total citations number	85 [53–114]	9 [2–20]	< 0.001
Publication year	2013 [2009–2017]	2016 [2013–2019]	< 0.001
Journal’s impact factor	5.3 [4.3–7.6]	4.3 [1.9–4.6]	< 0.001
Subject			0.001
Benign	16 (19.0%)	160 (21.2%)	
Endometriosis	2 (2.4%)	25 (3.3%)	
Fertility	4 (4.8%)	4 (0.5%)	
General	9 (10.7%)	85 (11.3%)	
Obstetrics	0 (0%)	8 (1.1%)	
Oncology	45 (53.6%)	299 (39.7%)	
Technique	1 (1.2%)	24 (3.2%)	
Urogynecology	7 (8.3%)	149 (19.8%)	
Continent			0.044
North America	62 (73.8%)	423 (56.1%)	
Europe	15 (17.9%)	203 (26.9%)	
Asia	6 (7.1%)	117 (15.5%)	
Australia	1 (1.2%)	4 (0.5%)	
South America	0 (0%)	6 (0.8%)	
Africa	0 (0%)	1 (0.1%)	
Country’s level of income			0.311
High	82 (97.6%)	706 (93.6%)	
Medium high	2 (2.4%)	39 (5.2%)	
Low medium	0 (0%)	9 (1.2%)	
Low	0 (0%)	0 (0%)	
Methodology			0.003
Retrospective	46 (54.8%)	348 (46.2%)	
Prospective	20 (24.8%)	157 (20.8%)	
Case report	1 (1.2%)	78 (10.3%)	
Review	7 (8.4%)	86 (11.4%)	
Case series	2 (2.4%)	42 (5.6%)	
Randomized controlled trial	7 (9.5%)	22 (2.9%)	
Video	0 (0%)	20 (2.7%)	
Meta-analysis	0 (0%)	1 (0.1%)	

Data are number (%) or median (interquartile range)

In a multivariable regression analysis (Table 4), including publication year, impact factor, subject of article, continent, and study design, the following factors were associated with a higher CPY: journal’s impact factor [adjusted odds ratio (aOR) 95% confidence interval (CI) 1.30 (1.16–1.41)], subject of study being oncology [aOR 95% CI 1.73 (1.06–2.81)] and randomized controlled trials [aOR 95% CI 3.67 (1.47–9.16)]. Publication year was negatively independently associated with a high CPY [aOR 95% CI 0.93 (0.88–0.98)].

**Table 4:** Multivariable regression analysis of predictors for a high citations per year score

Variable	Adjusted odds ratio (95% confidence interval)	p value
Publication year	0.93 (0.88–0.98)	0.018
Impact factor	1.30 (1.16–1.41)	< 0.001
Subject		0.027
Other	Ref	
Oncology	1.73 (1.06–2.81)	
Continent		0.096
Other	Ref	
North America	1.61 (0.91–2.84)	
Design		0.005
Other	Ref	
Randomized controlled trial	3.67 (1.47–9.16)	

### Timeline context

In a comparison of publications prior to 2015 vs. 2015 and later (Table 5), all median bibliometric scores were higher in the earlier period. The following areas of research had higher representation in the late period compared to the earlier period: fertility [8 (1.6%) vs. 0 (0%), respectively] and urogynecology [109 (22.4%) vs. 47 (13.4%), respectively]. After 2015 there has been a higher representation of publications from Asia [96 (19.7%) vs. 27 (7.7%)] and from LMIC [41 (8.4%) vs. 9 (2.6%)], a lower proportion of review articles [41 (8.4%) vs. 52 (14.8%)] and higher proportion of video publications [17 (3.5%) vs. 3 (0.9%)].

**Table 5:** Characteristics of included articles—publications before 2015 versus 2015 and later

Variable	< 2015 (n = 351)	≥ 2015 (n = 487)	p value
Citations per year	2.5 [1.1–4.6]	1.5 [0.4–3.0]	< 0.001
Relative citation ratio	1.3 [0.6–2.4]	0.6 [0.1–1.4]	< 0.001
Field citation ratio	3.1 [2.7–3.7]	2.9 [2.3–3.9]	< 0.001
Total citation number	27 [11–48]	5 [1–12]	< 0.001
Journal’s impact factor	4.5 [2.5–5.3]	4.2 [1.9–4.6]	< 0.001
Subject			< 0.001
Benign	81 (23.1%)	95 (19.5%)	
Endometriosis	9 (2.6%)	18 (3.7%)	
Fertility	0 (0%)	8 (1.6%)	
General	49 (14.0%)	45 (9.2%)	
Obstetrics	5 (1.4%)	3 (0.6%)	
Oncology	146 (41.6%)	198 (40.7%)	
Technique	14 (4.0%)	11 (2.3%)	
Urogynecology	47 (13.4%)	109 (22.4%)	
Continent			< 0.001
North America	245 (69.8%)	240 (49.3%)	
Europe	77 (21.9%)	141 (29.0%)	
Asia	27 (7.7%)	96 (19.7%)	
Australia	2 (0.6%)	3 (0.6%)	
South America	0 (0%)	6 (1.2%)	
Africa	0 (0%)	1 (0.2%)	
Country’s level of income			0.002
High	342 (97.4%)	446 (91.6%)	
Medium high	8 (2.3%)	33 (6.8%)	
Low medium	1 (0.3%)	8 (1.6%)	
Low	0 (0%)	0 (0%)	
Methodology			< 0.001
Retrospective	158 (45.0%)	236 (48.5%)	
Prospective	72 (20.5%)	105 (21.6%)	
Case reports	35 (10.0%)	44 (9.0%)	
Reviews	52 (14.8%)	41 (8.4%)	
Case series	22 (6.3%)	22 (4.5%)	
Randomized controlled trial	8 (2.3%)	22 (4.5%)	
Video	3 (0.9%)	17 (3.5%)	
Meta-analysis	1 (0.3%)	0 (0%)	

Data are number (%) or median [interquartile range]

## Discussion

The main findings of the current study underline that robotic surgery literature is dominated by publications of North American origin and from high income countries. Gynecologic oncology is the main subspecialty of research published and it is independently associated with high CPY score. Bibliometric scores of studies from LMIC are lower compared to high income countries and research quality from LMIC is lower.

### Results in context

Robotic surgery research emerged approximately two decades ago. Its implementation in gynecology has been a major source of robotic surgery academic literature [6]. The first use of robotic surgery in gynecology was for tubal anastomosis in 1998 [20]. In 2005 the FDA approved the use of robotic surgery in gynecology and since then it has become a common approach in gynecology [21], mainly oncology. In the past decade the use of robotic surgical systems for all kinds of gynecological and non‐gynecological surgery has increased [22] (Figure S2). It has been used for various gynecologic procedures including hysterectomy, myomectomy, sacrocolpopexy, endometriosis surgery, gynecologic cancer and more [21]. According to Intuitive Surgical, the manufacturer of the da Vinci robotic Surgical System, the most widely used robotic device worldwide, 6730 system has been installed worldwide as of December 2021 [23].

Gynecologic oncology was the most frequently studied topic in gynecologic robotic surgery in our bibliometric study. In a recent general robotic surgery scientific review, the top two gynecological journals publishing robotic surgery research were the Journal of Minimally Invasive Gynecology with 77 publications, a journal that publishes both benign gynecology and gynecologic oncology articles, and the journal Gynecologic Oncology with 53 publications [6]. Since the FDA’s approval of the da Vinci Surgical System for gynecologic procedures it has integrated rapidly into the surgical treatment of endometrial cancer. This process lead to substantial research on this topic associating its use with favorable outcomes [24]. Later, as the LACC trial raised controversy concerning the use of minimally invasive radical hysterectomy as the primary treatment for early stage cervical cancer [25], associated with a need for further ongoing large randomized trials regarding the role of robotics in this setting [26]. Furthermore, robotic surgery has recently been integrated into cytoreductive surgery in ovarian cancer [27, 28], further expanding the gynecologic oncology literature on robotic surgery. Importantly, the oncologic research importance and impact is reflected by our finding that it is independently associated with a high CPY score.

**Fig. 4:**
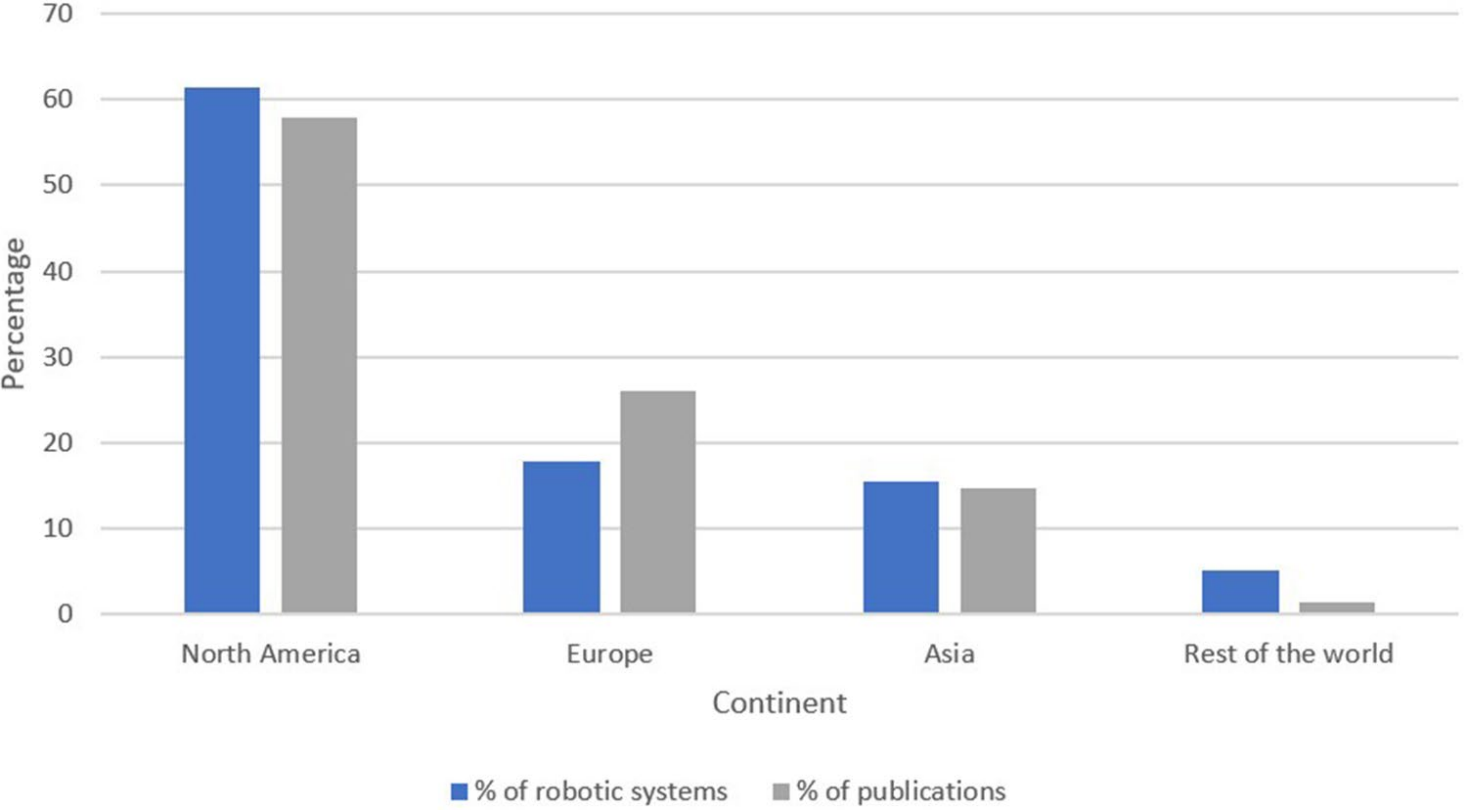
Proportions of da Vinci Surgical Systems and publications according to continent

We found that most publications originated in North America. According to Intuitive Surgical, 61.5% of the da Vinci systems installed until 2021 were in the U.S. The remaining systems were installed in Europe (17.8%), Asia (15.6%) and the rest of the world (5.1%). This report parallels the proportion of publications from different continents in our study (Fig. 4). Robotic surgery was originally developed to allow for remote surgery in space and for military purposes [29, 30]. Similarly, robotic surgery devices can potentially allow access to advanced surgery in remote areas, including low resource countries. Intuitive Surgical were the only providers in the global market for many years, and their hegemony is slowly decreasing with an 80% share in 2020 [31, 32]. Thus, this company’s report on the number of robotic surgical systems in LMIC, along with the proportion of publications from these countries, may provide a close approximation of their access and use of robot-assisted surgery. These data reveal a major disparity between high income countries and LMIC, that can be explained by the high estimated cost of robot-assisted laparoscopy systems, ranging $1.8–2.3 million U.S. dollars, the high maintenance costs, and the additional costs for the instruments, resulting in approximately $2000 additional U.S. dollars per robot-assisted case compared to laparotomy [33].

We did find an increase in the representation of LMIC during the recent period, corresponding to the recent introduction of robotic systems, leading to publications based on lower numbers, case series and case reports, and mostly on benign gynecology rather than oncologic diseases. Considering the potential benefits of robot-assisted surgery to patients and surgeons, this disparity raises the opportunity to improve quality of healthcare in LMIC.

The previous increase in the number of publications found in our study is in line with the ongoing increased rate of robotic surgery in gynecology. The rate of hysterectomies performed robotically has increased parallel to a decrease in abdominal hysterectomies, as well as conventional laparoscopic and vaginal hysterectomies, during the last two decades [34–36]. The rate of myomectomies performed robotically has also increased since 2012 [21]. According to Intuitive Surgical, gynecology is the second largest U.S. surgical specialty using robotic systems in 2021. The number of procedures performed grew from approximately 282,000 in 2019 to 316,000 in 2021 [23]. While the number of publications might not be an exact proxy of clinical use of robotic surgery, it may reflect its emergence as a dominant surgical approach. It is unclear why the number of publications per year reached a peak in 2014 and hasn’t grown since then. It is possible that studies report robotic procedures as part of a cohort of minimally invasive surgeries, together with conventional laparoscopy. Indeed, a recent scientific review of robotic surgery literature reported an ongoing increase in publications per year [6]. However, between 2014 and 2018 there was a smaller increase in the publication rate. Unfortunately, this trend is not discussed in that review. As robotic gynecologic surgery may be advantageous both to patients and surgeons, this literary “plateau” should be further investigated. Alternatively, the innovation introduced by robotics has plateaued and further new technologies associated with the computer interface, such as image analysis, machine learning, and artificial intelligence are anticipated in order to be researched and reported.

### Strengths and limitations

Our bibliometric study has some limitations inherent to this type of studies. First, we used one literature database and may have missed robotic surgery studies. Second, we reported impact factor per journal according to the latest available impact factor list, and not by publication year’s specific impact factor. This may introduce skewing of the results. Furthermore, a topic-specific limitation is that articles studying robotic surgery may not be titled as such and thus were not included in our analysis. However, the selection of studies that focus on robotic surgery makes our bibliometric representation of the literature more specific.

We did not find a prior study evaluating publications and bibliometrics of robotic surgery in OBGYN. Thus, this study may be the first evaluation of this research question. Second, we used several citation metrics in our analysis, including CPY and RCR, which overcome the bias of counting the total citations number, favoring older studies.

## Conclusion

Robotic surgery publication rate in OBGYN reached a peak nearly a decade ago. Effort should be made to reinstitute academic momentum. The origin of most of the studies is high income countries and research quality from LMIC is awaited. These results provide an incentive to stimulate access of LMIC to high quality healthcare resources such as robotic surgery.

## Supplementary Information

Below is the link to the electronic supplementary material.

Supplementary file1 Figure S1 Number of articles published per 5 years. (JPG 54 KB)

Supplementary file2 Figure S2 Number of articles published by year according to medical discipline. (JPG 81 KB)

## Data Availability

The data that support the findings of this study are available from the corresponding author, RM, upon reasonable request.
