# Supplementary figures and images for: Robotic surgery in obstetrics and gynecology: a bibliometric study
Source: J Robot Surg. 2023 Jul 10;17(5):2387–97. doi: 10.1007/s11701-023-01672-1 (PMC10492767; doi:10.1007/s11701-023-01672-1)

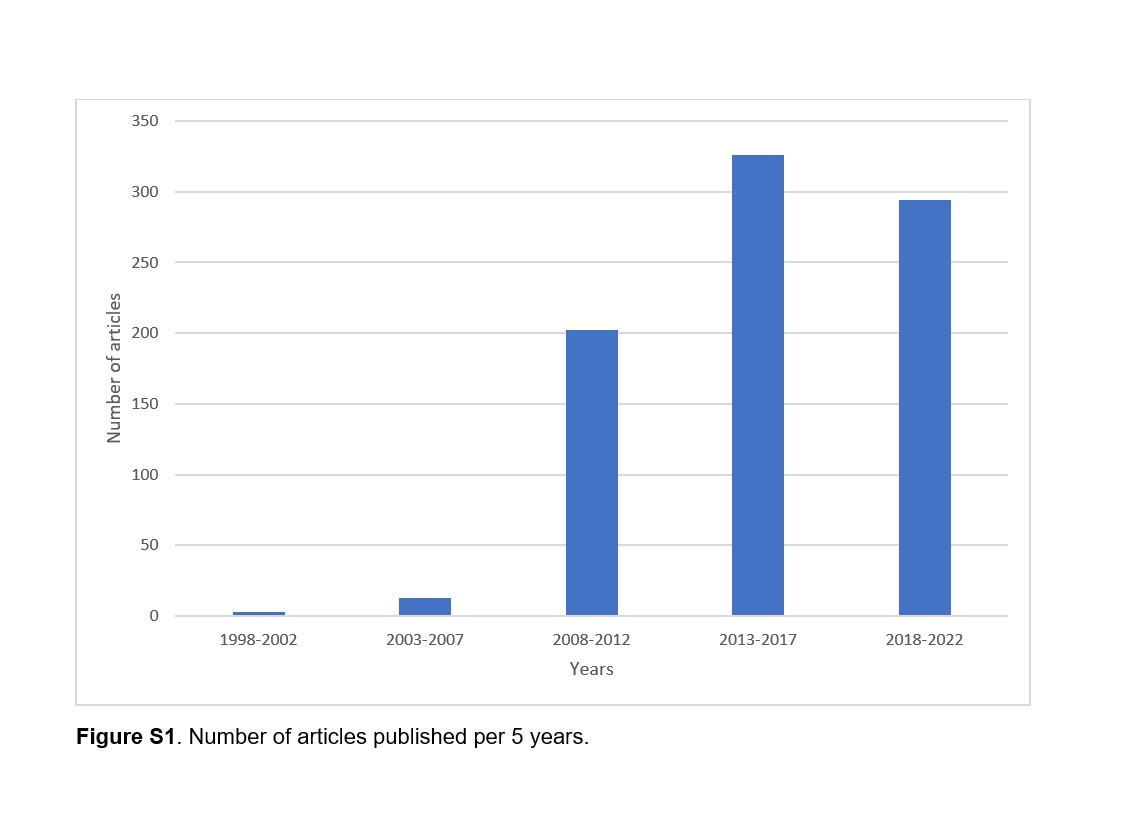

Supplement: Supplementary file 1 — Supplementary file1 Figure S1 Number of articles published per 5 years. (JPG 54 KB) [file 11701_2023_1672_MOESM1_ESM.jpg]

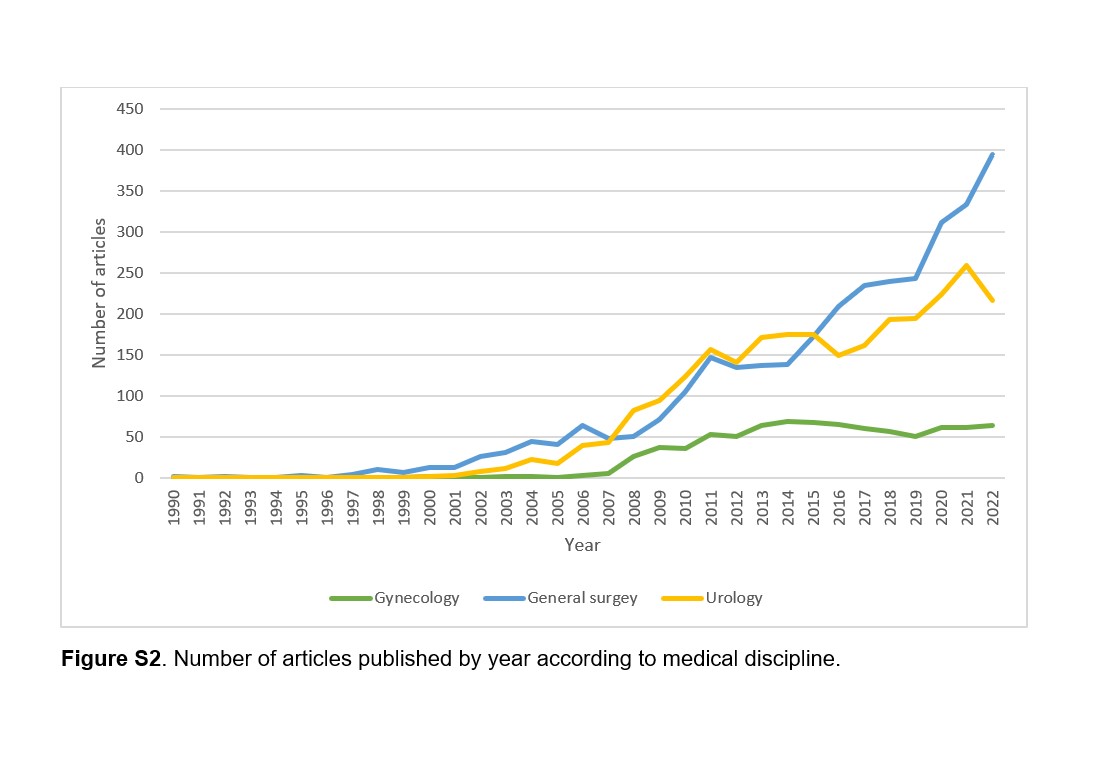

Supplement: Supplementary file 2 — Supplementary file2 Figure S2 Number of articles published by year according to medical discipline. (JPG 81 KB) [file 11701_2023_1672_MOESM2_ESM.jpg]
